# 1,4-Diferrocenyl-2-methyl­piperazine-1,4-diium bis­(trifluoro­acetate)

**DOI:** 10.1107/S160053680902426X

**Published:** 2009-07-22

**Authors:** Fang Chen

**Affiliations:** a Ordered Matter Science Research Center, College of Chemistry and Chemical Engineering, Southeast University, Nanjing 210096, People’s Republic of China

## Abstract

In the title compound, [Fe_2_(C_5_H_5_)_2_(C_17_H_24_N_2_)](CF_3_COO)_2_, the cation possesses a crystallographically imposed inversion centre. The methyl group is disordered over two positions of equal occupancy. The Fe—C bond lengths to the two cyclo­penta­diene rings vary from 2.025 (6) to 2.044 (6) Å. Inter­molecular N—H⋯O and C—H⋯O hydrogen bonds link the cations and anions into a three-dimensional network.

## Related literature

For the applications of ferrocene derivatives, see: Yang *et al.* (2002 ▶); Togni & Hayashi (1995 ▶); Long (1995 ▶); Roberto *et al.* (2000 ▶). For the crystal structure of related compounds, see: Hess *et al.* (1999 ▶); Base *et al.* (2002 ▶); For the synthetic strategy, see: Chen (2009 ▶).
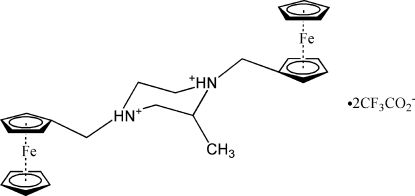


## Experimental

### 

#### Crystal data

[Fe_2_(C_5_H_5_)_2_(C_17_H_24_N_2_)](C_2_F_3_O_2_)_2_
*M* *_r_* = 724.30
Monoclinic, 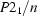

*a* = 11.922 (3) Å
*b* = 9.7977 (16) Å
*c* = 13.628 (4) Å
β = 99.998 (15)°
*V* = 1567.7 (7) Å^3^
*Z* = 2
Mo *K*α radiation
μ = 1.00 mm^−1^
*T* = 293 K
0.27 × 0.25 × 0.20 mm

#### Data collection

Rigaku SCXmini diffractometer
Absorption correction: multi-scan (*CrystalClear*; Rigaku, 2005 ▶) *T*_min_ = 0.771, *T*_max_ = 0.819
15611 measured reflections
3592 independent reflections
3117 reflections with *I* > 2σ(*I*)
*R*_int_ = 0.042

#### Refinement

*R*[*F* ^2^ > 2σ(*F* ^2^)] = 0.078
*wR*(*F* ^2^) = 0.224
*S* = 1.07
3592 reflections
209 parameters
H-atom parameters constrained
Δρ_max_ = 1.06 e Å^−3^
Δρ_min_ = −0.71 e Å^−3^

### 

Data collection: *CrystalClear* (Rigaku, 2005 ▶); cell refinement: *CrystalClear*; data reduction: *CrystalClear*; program(s) used to solve structure: *SHELXS97* (Sheldrick, 2008 ▶); program(s) used to refine structure: *SHELXL97* (Sheldrick, 2008 ▶); molecular graphics: *SHELXTL* (Sheldrick, 2008 ▶); software used to prepare material for publication: *SHELXL97*.

## Supplementary Material

Crystal structure: contains datablocks I, global. DOI: 10.1107/S160053680902426X/rz2338sup1.cif

Structure factors: contains datablocks I. DOI: 10.1107/S160053680902426X/rz2338Isup2.hkl

Additional supplementary materials:  crystallographic information; 3D view; checkCIF report

## Figures and Tables

**Table 1:** Hydrogen-bond geometry (Å, °)

*D*—H⋯*A*	*D*—H	H⋯*A*	*D*⋯*A*	*D*—H⋯*A*
N1—H1*A*⋯O1^i^	0.91	1.80	2.696 (6)	169
C4—H4⋯O2^ii^	0.98	2.35	3.306 (9)	163

Symmetry codes: (i) 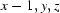; (ii) 
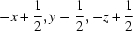
.

**Fig. 1.**
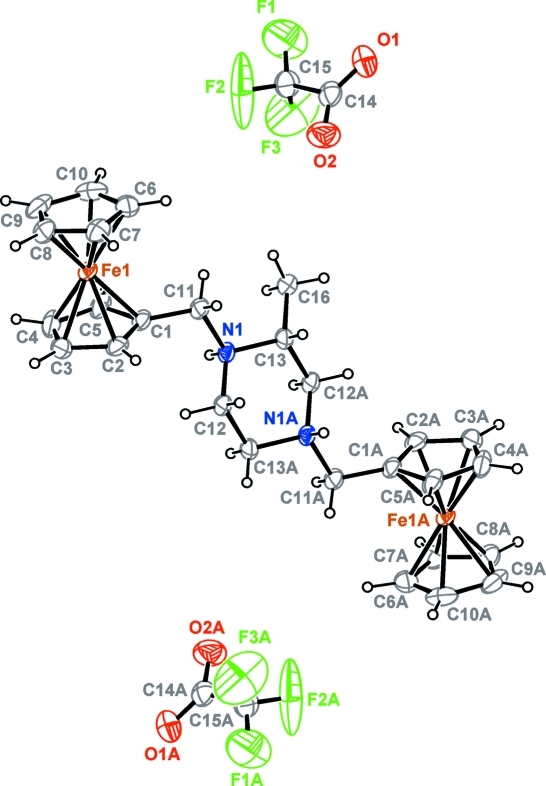
The structure of the title compound with displacement ellipsoids drawn at the 30% probability level. Atoms labelled with the suffix A are generated by the symmetry operator (-x, 1-y, 1-z). Only one component of the disordered methyl group is shown.

**Fig. 2.**
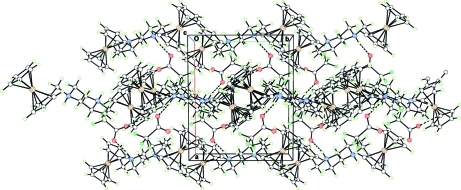
Packing diagram of the title compound viewed along the c axis. Intermolecular hydrogen bonds are shown as dashed lines. Only one component of the disordered methyl group is shown.

## supplementary crystallographic information

### Comment

The chemistry of ferrocene has received much attention because of its applications in many fields, such as in catalysis (Yang *et al.*, 2002), non-linear optical (NLO) materials (Long, 1995; Roberto *et al.*, 2000), organic or organometallic synthesis and materials (Togni & Hayashi, 1995), and so on. As part of our on-going studies on new ferrocene compounds, the crystal structure of the title compound is reported herein.

The title compound (Fig. 1) consists of centrosymmetric 1,4-ferrocenyl-2-methylpiperazinium cations and trifluoroacetate anions in the stroichiometric ratio of 1:2. The methyl group of the cation is disordered over two positions of equal occupancy related by the symmetry operator (-x, 1-y, 1-z). The deformation of the 1,4-ferrocenyl-2-methylpiperazinium cation is reflected in the values of the C12A—C13—N1, C13—N1—C12, N1—C12—C13A angles, which are 109.8 (4), 110.0 (3), 111.9 (4)°, respectively [symmetry code: (A) -x, 1-y, 1-z]. The Fe—C distances to the two cyclopentadiene rings are normal, ranging from 2.025 (6) to 2.044 (6) Å (Hess *et al.*, 1999; Base *et al.*, 2002). The two cyclopentadiene rings are nearly parallel, forming a dihedral angle of 1.1 (2)°. In the crystal packing (Fig. 2), intermolecular N—H···O and C—H···O interactions (Table 1) link cations and anions into a hydrogen-bonded network, which stabilizes the crystal packing (Fig.2).

### Experimental

The preparation of *S*-1,4-ferrocenyl-2-methylpiperazine is analogous to that of 2,2'-diferrocenyl-5,5'-(*m*-phenylene)di-2*H*-tetrazole (Chen, 2009). To a mixture of [Fe(C_5_H_5_)(C_5_H_4_)N^+^(CH_3_)_3_I^-^] (10 mmol) in H_2_O (50 ml) was added *S*-2-methylpiperazine (5 mmol) and the mixture was heated to reflux temperature for 5 h. Then, the formed precipitate was filtered, the obtained yellow solid was purified enough without further disposal (yield: 78%). For the preparation of the title compound, a solution of trifluoroacetic acid (4 mmol) in ethanol was added to a solution of *S*-1,4-ferrocenyl-2-methylpiperazine (2 mmol) in dichloromethane/ethanol (1:1 v/v) and the mixture stirred for 1 h at room temperature. Red crystals suitable for X-ray diffraction analysis were obtained by slow evaporation of the solution at room temperature after 5 days.

### Refinement

Positional parameters of all the H atoms were calculated geometrically and were allowed to ride on the parent atoms, with C—H = 0.96-0.98 Å, N—H = 0.91 Å and with *U*_iso_(H) = 1.2*U*_iso_(C, N) or 1.2*U*_iso_(C) for methyl H atoms. The methyl group is disordered over two positions related by a centre of symmetry, with site occupancy factors of 0.5.

### Crystal data

**Table:**

[Fe_2_(C_5_H_5_)_2_(C_17_H_24_N_2_)](C_2_F_3_O_2_)_2_	*F*(000) = 744.0
*M**_r_* = 724.30	*D*_x_ = 1.534 Mg m^−^^3^
Monoclinic, *P*2_1_/*n*	Mo *K*α radiation, λ = 0.71073 Å
Hall symbol: -P 2yn	Cell parameters from 3933 reflections
*a* = 11.922 (3) Å	θ = 2.6–27.5°
*b* = 9.7977 (16) Å	µ = 1.00 mm^−^^1^
*c* = 13.628 (4) Å	*T* = 293 K
β = 99.998 (15)°	Block, red
*V* = 1567.7 (7) Å^3^	0.27 × 0.25 × 0.20 mm
*Z* = 2	

### Data collection

**Table:**

Rigaku SCXmini diffractometer	3592 independent reflections
Radiation source: fine-focus sealed tube	3117 reflections with *I* > 2σ(*I*)
graphite	*R*_int_ = 0.042
Detector resolution: 13.6612 pixels mm^-1^	θ_max_ = 27.5°, θ_min_ = 2.5°
ω scans	*h* = −15→15
Absorption correction: multi-scan (*CrystalClear*; Rigaku, 2005)	*k* = −12→12
*T*_min_ = 0.771, *T*_max_ = 0.819	*l* = −17→17
15611 measured reflections	

### Refinement

**Table:**

Refinement on *F*^2^	Primary atom site location: structure-invariant direct methods
Least-squares matrix: full	Secondary atom site location: difference Fourier map
*R*[*F*^2^ > 2σ(*F*^2^)] = 0.078	Hydrogen site location: inferred from neighbouring sites
*wR*(*F*^2^) = 0.224	H-atom parameters constrained
*S* = 1.07	*w* = 1/[σ^2^(*F*_o_^2^) + (0.1088*P*)^2^ + 3.5578*P*] where *P* = (*F*_o_^2^ + 2*F*_c_^2^)/3
3592 reflections	(Δ/σ)_max_ < 0.001
209 parameters	Δρ_max_ = 1.06 e Å^−^^3^
0 restraints	Δρ_min_ = −0.70 e Å^−^^3^

### Special details

**Table:**

Geometry. All e.s.d.'s (except the e.s.d. in the dihedral angle between two l.s. planes) are estimated using the full covariance matrix. The cell e.s.d.'s are taken into account individually in the estimation of e.s.d.'s in distances, angles and torsion angles; correlations between e.s.d.'s in cell parameters are only used when they are defined by crystal symmetry. An approximate (isotropic) treatment of cell e.s.d.'s is used for estimating e.s.d.'s involving l.s. planes.
Refinement. Refinement of *F*^2^ against ALL reflections. The weighted *R*-factor *wR* and goodness of fit *S* are based on *F*^2^, conventional *R*-factors *R* are based on *F*, with *F* set to zero for negative *F*^2^. The threshold expression of *F*^2^ > σ(*F*^2^) is used only for calculating *R*-factors(gt) *etc*. and is not relevant to the choice of reflections for refinement. *R*-factors based on *F*^2^ are statistically about twice as large as those based on *F*, and *R*- factors based on ALL data will be even larger.

### Fractional atomic coordinates and isotropic or equivalent isotropic displacement parameters (Å^2^)

**Table:**

	*x*	*y*	*z*	*U*_iso_*/*U*_eq_	Occ. (<1)
Fe1	0.07028 (5)	0.09197 (7)	0.20528 (5)	0.0426 (3)	
N1	0.0206 (3)	0.3711 (4)	0.4525 (3)	0.0398 (8)	
H1A	−0.0408	0.3234	0.4651	0.048*	
C1	0.0197 (4)	0.2288 (5)	0.3010 (3)	0.0451 (10)	
C13	0.0910 (4)	0.4133 (5)	0.5509 (4)	0.0488 (11)	
H13A	0.1587	0.4611	0.5394	0.059*	
H13B	0.1147	0.3329	0.5907	0.059*	0.50
C7	0.2223 (5)	0.0916 (6)	0.1564 (5)	0.0591 (13)	
H7	0.2594	0.1718	0.1333	0.071*	
C12	−0.0211 (4)	0.4941 (5)	0.3933 (3)	0.0449 (10)	
H12A	−0.0675	0.4657	0.3310	0.054*	
H12B	0.0435	0.5447	0.3777	0.054*	
C11	0.0902 (4)	0.2780 (5)	0.3958 (3)	0.0469 (10)	
H11A	0.1556	0.3276	0.3809	0.056*	
H11B	0.1178	0.2004	0.4372	0.056*	
C8	0.1457 (5)	0.0064 (6)	0.0967 (4)	0.0614 (14)	
H8	0.1201	0.0160	0.0247	0.074*	
C5	−0.0521 (5)	0.1107 (6)	0.2920 (4)	0.0583 (14)	
H5	−0.0641	0.0495	0.3462	0.070*	
C2	0.0127 (5)	0.2876 (6)	0.2044 (4)	0.0569 (13)	
H2	0.0527	0.3694	0.1876	0.068*	
C6	0.2371 (5)	0.0449 (7)	0.2551 (5)	0.0642 (15)	
H6	0.2864	0.0864	0.3123	0.077*	
C9	0.1104 (6)	−0.0962 (6)	0.1582 (6)	0.0747 (19)	
H9	0.0572	−0.1710	0.1367	0.090*	
C3	−0.0642 (5)	0.2062 (7)	0.1373 (5)	0.0707 (18)	
H3	−0.0851	0.2215	0.0654	0.085*	
C4	−0.1032 (5)	0.0992 (7)	0.1902 (6)	0.075 (2)	
H4	−0.1560	0.0272	0.1616	0.090*	
C10	0.1680 (6)	−0.0701 (7)	0.2578 (5)	0.0748 (19)	
H10	0.1612	−0.1240	0.3170	0.090*	
O1	0.8567 (4)	0.2055 (6)	0.4984 (3)	0.0771 (13)	
O2	0.7403 (5)	0.3147 (6)	0.3831 (5)	0.1058 (19)	
C15	0.6838 (6)	0.1017 (7)	0.4333 (6)	0.0715 (17)	
C14	0.7705 (5)	0.2200 (7)	0.4397 (5)	0.0664 (15)	
F2	0.6434 (12)	0.0677 (12)	0.3507 (5)	0.283 (8)	
F3	0.6053 (8)	0.1223 (11)	0.4759 (11)	0.257 (6)	
F1	0.7170 (8)	−0.0077 (8)	0.4721 (10)	0.240 (6)	
C16	0.1208 (9)	0.2777 (11)	0.6216 (8)	0.056 (2)	0.50
H16A	0.0515	0.2310	0.6276	0.084*	0.50
H16B	0.1590	0.3051	0.6865	0.084*	0.50
H16C	0.1692	0.2177	0.5920	0.084*	0.50

### Atomic displacement parameters (Å^2^)

**Table:**

	*U*^11^	*U*^22^	*U*^33^	*U*^12^	*U*^13^	*U*^23^
Fe1	0.0450 (4)	0.0407 (4)	0.0431 (4)	0.0050 (3)	0.0104 (3)	−0.0119 (3)
N1	0.0385 (18)	0.0455 (19)	0.0358 (17)	−0.0006 (15)	0.0072 (14)	−0.0136 (15)
C1	0.047 (2)	0.044 (2)	0.046 (2)	0.0033 (19)	0.0123 (19)	−0.0150 (19)
C13	0.045 (2)	0.057 (3)	0.042 (2)	0.005 (2)	−0.0001 (18)	−0.015 (2)
C7	0.055 (3)	0.060 (3)	0.068 (3)	0.001 (2)	0.027 (3)	−0.010 (3)
C12	0.050 (2)	0.048 (2)	0.037 (2)	0.003 (2)	0.0074 (18)	−0.0122 (19)
C11	0.047 (2)	0.053 (3)	0.042 (2)	0.005 (2)	0.0113 (18)	−0.014 (2)
C8	0.067 (3)	0.070 (3)	0.051 (3)	0.009 (3)	0.019 (2)	−0.020 (3)
C5	0.055 (3)	0.057 (3)	0.069 (3)	−0.007 (2)	0.025 (3)	−0.027 (3)
C2	0.073 (3)	0.048 (3)	0.049 (3)	0.020 (2)	0.010 (2)	−0.010 (2)
C6	0.052 (3)	0.072 (4)	0.067 (3)	0.016 (3)	0.006 (2)	−0.013 (3)
C9	0.075 (4)	0.042 (3)	0.113 (6)	0.002 (3)	0.031 (4)	−0.024 (3)
C3	0.072 (4)	0.078 (4)	0.054 (3)	0.030 (3)	−0.010 (3)	−0.024 (3)
C4	0.041 (3)	0.086 (4)	0.096 (5)	−0.003 (3)	0.006 (3)	−0.056 (4)
C10	0.090 (5)	0.061 (4)	0.079 (4)	0.035 (3)	0.030 (4)	0.021 (3)
O1	0.063 (2)	0.113 (4)	0.057 (2)	−0.015 (2)	0.017 (2)	−0.009 (2)
O2	0.104 (4)	0.068 (3)	0.138 (5)	0.003 (3)	0.001 (4)	0.025 (3)
C15	0.063 (4)	0.069 (4)	0.082 (4)	−0.015 (3)	0.013 (3)	−0.001 (3)
C14	0.063 (3)	0.076 (4)	0.062 (3)	0.004 (3)	0.018 (3)	−0.021 (3)
F2	0.427 (15)	0.321 (12)	0.086 (4)	−0.306 (13)	0.004 (7)	−0.023 (6)
F3	0.156 (7)	0.224 (10)	0.436 (18)	−0.078 (7)	0.181 (10)	−0.056 (11)
F1	0.200 (8)	0.099 (5)	0.378 (15)	−0.057 (5)	−0.075 (9)	0.070 (7)
C16	0.061 (6)	0.050 (5)	0.053 (5)	0.008 (5)	0.002 (4)	−0.012 (4)

### Geometric parameters (Å, °)

**Table:**

Fe1—C10	2.025 (6)	C12—H12B	0.9700
Fe1—C1	2.034 (4)	C11—H11A	0.9700
Fe1—C2	2.035 (5)	C11—H11B	0.9700
Fe1—C7	2.036 (5)	C8—C9	1.419 (9)
Fe1—C9	2.036 (5)	C8—H8	0.9800
Fe1—C6	2.039 (6)	C5—C4	1.420 (9)
Fe1—C5	2.040 (5)	C5—H5	0.9800
Fe1—C8	2.041 (5)	C2—C3	1.422 (8)
Fe1—C3	2.041 (6)	C2—H2	0.9800
Fe1—C4	2.044 (6)	C6—C10	1.400 (10)
N1—C12	1.487 (6)	C6—H6	0.9800
N1—C13	1.511 (5)	C9—C10	1.433 (10)
N1—C11	1.529 (5)	C9—H9	0.9800
N1—H1A	0.9100	C3—C4	1.397 (10)
C1—C2	1.426 (7)	C3—H3	0.9800
C1—C5	1.432 (7)	C4—H4	0.9800
C1—C11	1.493 (6)	C10—H10	0.9800
C13—C12^i^	1.521 (6)	O1—C14	1.197 (8)
C13—C16	1.643 (12)	O2—C14	1.220 (8)
C13—H13A	0.9700	C15—F2	1.192 (9)
C13—H13B	0.9700	C15—F3	1.201 (11)
C7—C8	1.391 (8)	C15—F1	1.229 (10)
C7—C6	1.402 (9)	C15—C14	1.546 (9)
C7—H7	0.9800	C16—H16A	0.9600
C12—C13^i^	1.521 (6)	C16—H16B	0.9600
C12—H12A	0.9700	C16—H16C	0.9600
			
C10—Fe1—C1	120.4 (3)	C13^i^—C12—H12A	109.2
C10—Fe1—C2	155.9 (3)	N1—C12—H12B	109.2
C1—Fe1—C2	41.0 (2)	C13^i^—C12—H12B	109.2
C10—Fe1—C7	67.9 (3)	H12A—C12—H12B	107.9
C1—Fe1—C7	126.2 (2)	C1—C11—N1	110.9 (4)
C2—Fe1—C7	108.6 (2)	C1—C11—H11A	109.5
C10—Fe1—C9	41.3 (3)	N1—C11—H11A	109.5
C1—Fe1—C9	155.6 (3)	C1—C11—H11B	109.5
C2—Fe1—C9	161.5 (3)	N1—C11—H11B	109.5
C7—Fe1—C9	67.9 (3)	H11A—C11—H11B	108.0
C10—Fe1—C6	40.3 (3)	C7—C8—C9	108.1 (5)
C1—Fe1—C6	108.4 (2)	C7—C8—Fe1	69.9 (3)
C2—Fe1—C6	121.5 (3)	C9—C8—Fe1	69.5 (3)
C7—Fe1—C6	40.2 (2)	C7—C8—H8	125.9
C9—Fe1—C6	68.4 (3)	C9—C8—H8	125.9
C10—Fe1—C5	106.9 (3)	Fe1—C8—H8	125.9
C1—Fe1—C5	41.2 (2)	C4—C5—C1	107.2 (6)
C2—Fe1—C5	69.1 (2)	C4—C5—Fe1	69.8 (3)
C7—Fe1—C5	163.1 (2)	C1—C5—Fe1	69.2 (3)
C9—Fe1—C5	119.7 (3)	C4—C5—H5	126.4
C6—Fe1—C5	125.6 (3)	C1—C5—H5	126.4
C10—Fe1—C8	68.6 (3)	Fe1—C5—H5	126.4
C1—Fe1—C8	162.4 (2)	C3—C2—C1	107.2 (6)
C2—Fe1—C8	125.0 (2)	C3—C2—Fe1	69.8 (3)
C7—Fe1—C8	39.9 (2)	C1—C2—Fe1	69.4 (3)
C9—Fe1—C8	40.8 (3)	C3—C2—H2	126.4
C6—Fe1—C8	67.8 (2)	C1—C2—H2	126.4
C5—Fe1—C8	155.1 (2)	Fe1—C2—H2	126.4
C10—Fe1—C3	161.6 (3)	C10—C6—C7	108.1 (6)
C1—Fe1—C3	68.5 (2)	C10—C6—Fe1	69.3 (3)
C2—Fe1—C3	40.8 (2)	C7—C6—Fe1	69.8 (3)
C7—Fe1—C3	121.7 (3)	C10—C6—H6	125.9
C9—Fe1—C3	124.2 (3)	C7—C6—H6	125.9
C6—Fe1—C3	156.7 (3)	Fe1—C6—H6	125.9
C5—Fe1—C3	68.2 (3)	C8—C9—C10	106.9 (6)
C8—Fe1—C3	107.8 (2)	C8—C9—Fe1	69.8 (3)
C10—Fe1—C4	124.9 (3)	C10—C9—Fe1	68.9 (3)
C1—Fe1—C4	68.5 (2)	C8—C9—H9	126.5
C2—Fe1—C4	68.4 (3)	C10—C9—H9	126.5
C7—Fe1—C4	155.5 (3)	Fe1—C9—H9	126.5
C9—Fe1—C4	106.8 (3)	C4—C3—C2	108.9 (5)
C6—Fe1—C4	162.3 (3)	C4—C3—Fe1	70.1 (3)
C5—Fe1—C4	40.7 (3)	C2—C3—Fe1	69.4 (3)
C8—Fe1—C4	120.5 (2)	C4—C3—H3	125.6
C3—Fe1—C4	40.0 (3)	C2—C3—H3	125.6
C12—N1—C13	110.0 (3)	Fe1—C3—H3	125.6
C12—N1—C11	111.6 (3)	C3—C4—C5	108.7 (5)
C13—N1—C11	110.2 (3)	C3—C4—Fe1	69.9 (3)
C12—N1—H1A	108.3	C5—C4—Fe1	69.5 (3)
C13—N1—H1A	108.3	C3—C4—H4	125.7
C11—N1—H1A	108.3	C5—C4—H4	125.7
C2—C1—C5	108.0 (5)	Fe1—C4—H4	125.7
C2—C1—C11	127.0 (5)	C6—C10—C9	107.8 (6)
C5—C1—C11	125.0 (5)	C6—C10—Fe1	70.4 (3)
C2—C1—Fe1	69.5 (3)	C9—C10—Fe1	69.8 (3)
C5—C1—Fe1	69.7 (3)	C6—C10—H10	126.1
C11—C1—Fe1	125.7 (3)	C9—C10—H10	126.1
N1—C13—C12^i^	109.8 (4)	Fe1—C10—H10	126.1
N1—C13—C16	109.2 (5)	F2—C15—F3	106.4 (11)
C12^i^—C13—C16	105.7 (5)	F2—C15—F1	102.1 (10)
N1—C13—H13A	109.7	F3—C15—F1	99.1 (10)
C12^i^—C13—H13A	109.7	F2—C15—C14	114.8 (7)
C16—C13—H13A	112.6	F3—C15—C14	114.6 (7)
N1—C13—H13B	109.7	F1—C15—C14	117.8 (7)
C12^i^—C13—H13B	109.7	O1—C14—O2	129.7 (7)
H13A—C13—H13B	108.2	O1—C14—C15	115.9 (7)
C8—C7—C6	109.0 (5)	O2—C14—C15	114.4 (6)
C8—C7—Fe1	70.2 (3)	C13—C16—H16A	109.5
C6—C7—Fe1	70.0 (3)	C13—C16—H16B	109.5
C8—C7—H7	125.5	H16A—C16—H16B	109.5
C6—C7—H7	125.5	C13—C16—H16C	109.5
Fe1—C7—H7	125.5	H16A—C16—H16C	109.5
N1—C12—C13^i^	111.9 (4)	H16B—C16—H16C	109.5
N1—C12—H12A	109.2		

Symmetry codes: (i) −*x*, −*y*+1, −*z*+1.

### Hydrogen-bond geometry (Å, °)

**Table:**

*D*—H···*A*	*D*—H	H···*A*	*D*···*A*	*D*—H···*A*
N1—H1A···O1^ii^	0.91	1.80	2.696 (6)	169
C4—H4···O2^iii^	0.98	2.35	3.306 (9)	163

Symmetry codes: (ii) *x*−1, *y*, *z*; (iii) −*x*+1/2, *y*−1/2, −*z*+1/2.
